# Evaluation of Intergovernmental Panel on Climate Change (IPCC) equations to predict enteric methane emission from lactating cows fed Mediterranean diets

**DOI:** 10.3168/jdsc.2022-0240

**Published:** 2023-02-09

**Authors:** S. Colombini, A. Rota Graziosi, G. Galassi, G. Gislon, G.M. Crovetto, D. Enriquez-Hidalgo, L. Rapetti

**Affiliations:** 1 Dipartimento di Scienze Agrarie e Ambientali, Università degli Studi di Milano, Milano 20133, Italy; 2 Sustainable Agriculture Sciences Department, University of Bristol, Bristol BS8 1TH, United Kingdom; 3 Rothamsted Research, Sustainable Agriculture Sciences, North Wyke, Okehampton, Devon EX20 2SB, United Kingdom

## Abstract

The study aimed to evaluate Intergovernmental Panel on Climate Change (IPCC) Tier 2 (2006 and 2019) to predict enteric CH_4_ emissions from lactating cows fed Mediterranean diets. The effects of the CH_4_ conversion factor (Y_m_; CH_4_ energy loss as a percentage of gross energy intake) and digestible energy (DE) of the diet were evaluated as model predictors. A data set was created using individual observations derived from 3 in vivo studies on lactating dairy cows housed in respiration chambers and fed diets typical of the Mediterranean region based on silages and hays. Five models using different Y_m_ and DE were evaluated following a Tier 2 approach: (1) average values of Y_m_ (6.5%) and DE (70%) from IPCC (2006); (2) average value of Y_m_ (5.7%) and DE (70.0%) from IPCC (2019; 1YM); (3) Y_m_ = 5.7% and DE measured in vivo (1YMIV); (4) Y_m_ = 5.7 or 6.0%, depending on dietary NDF, and DE = 70% (2YM); and (5) Y_m_ = 5.7 or 6.0%, depending on dietary NDF, and DE measured in vivo (2YMIV). Finally, a Tier 2 model for Mediterranean diets (MED) was derived from the Italian data set (Y_m_ = 5.58%; DE = 69.9% for silage-based diets and 64.8% for hay-based diets) and validated on an independent data set of cows fed Mediterranean diets. The most accurate models tested were 2YMIV, 2YM, and 1YMIV with predictions of 384, 377, and 377 (g of CH_4_/d), respectively, versus the in vivo value of 381. The most precise model was 1YM (slope bias = 1.88%; r = 0.63). Overall, 1YM showed the highest concordance correlation coefficient value (0.579), followed by 1YMIV (0.569). Cross-validation on an independent data set of cows fed Mediterranean diets (corn silage and alfalfa hay) resulted in concordance correlation coefficient of 0.492 and 0.485 for 1YM and MED, respectively. The prediction of MED (397) was more accurate than 1YM (405) in comparison with the corresponding in vivo value of 396 g of CH_4_/d. The results of this study showed that the average values proposed by IPCC (2019) can adequately predict CH_4_ emissions from cows fed typical Mediterranean diets. However, the use of specific factors for the Mediterranean area, such as DE, improved the accuracy of the models.

## Highlights

• The IPCC 2019 Tier 2 equations predict methane emission adequately.
• The knowledge of DE improves the accuracy of CH_4_ prediction.
• Hay-based diets reduce digestibility, and consequently, a measured DE should be applied.
• Local values of the CH_4_ conversion factor (Y_m_) and DE are proposed for the Mediterranean area.

## Graphical Abstract

**Figure:**
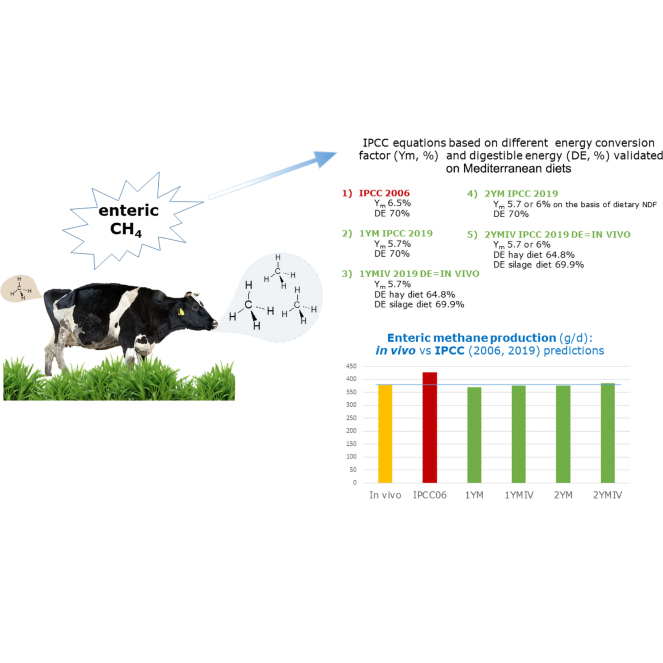
**Summary:** Intergovernmental Panel on Climate Change (IPCC) equations were evaluated to study the precision and accuracy in predicting methane (CH_4_) emission from lactating dairy cows. The predicted values were compared with in vivo values obtained from experimental studies conducted using cows fed diets based on hay or silage forages in the Mediterranean region. Hay-based diets differed significantly from silage-based diets having lower energy digestibility (DE). The IPCC 2019 equations predicted CH_4_ emission well and hence may be used for the computation of greenhouse gas inventories. However, specific equations are proposed to predict CH_4_ emission by dairy cows in the Mediterranean region.

Livestock and manure management contribute to 5.8% of anthropogenic-caused greenhouse gas (**GHG**) emissions, and considering CH_4_ emissions only, enteric fermentation and manure management represent 32% of anthropic CH_4_ emissions (World Resources Institute, 2019). Therefore, it is important to predict the amount of enteric CH_4_ emitted in a specific livestock production system (Niu et al., 2018). The Intergovernmental Panel on Climate Change (**IPCC**) guidelines for GHG inventories were developed to provide internationally agreed methodologies for estimating GHG emissions (IPCC, 2006a). The latest version of IPCC (2019b) was published to refine the previous version (IPCC, 2006b), providing supplementary methodologies and updated default values. Regarding enteric CH_4_ emission from ruminants, the conversion factor of gross energy intake into enteric CH_4_ energy (**Y_m_**) can be chosen according to the level of productivity and diet characteristics (IPCC, 2019b). The criteria to choose the value of Y_m_ are milk production, dietary NDF concentration, and digestible energy (**DE**, as % of GE intake). For example, considering the whole data set, IPCC (2019a) suggests a value of Y_m_ equal to 5.7% for high-yielding cows (>8,500 kg of milk/head/yr^−1^), associated with DE ≥70% and NDF ≤35% of diet DM. However, for this production level, the proposed values of NDF and Y_m_ may not represent the diets used in regions with Mediterranean climate characteristics. The Y_m_ values in IPCC (2019a) were based on the data set of Niu et al. (2018), which included 154 studies, 82 of them conducted in European research institutes, but none conducted in southern European countries. Diets from northern Europe (based on ryegrass and corn silages, March et al., 2014) are different from the diets of the Mediterranean region, since the latter are widely based on corn silage and alfalfa/grass hays (Gislon et al., 2020a). For this reason, IPCC (2019a) encourages the development of country-specific Y_m_ factors for a more accurate estimation of enteric CH_4_. The present study aimed to (1) compare the CH_4_ emissions estimated with IPCC (2019a) and IPCC (2006b) equations with the values obtained in vivo by cows fed typical Mediterranean diets; (2) develop possible improvements for the IPCC parameters (Y_m_ and DE) for a more accurate prediction of CH_4_ emission from lactating cows fed Mediterranean diets.

A data set of 66 individual observations was created to evaluate different IPCC equations to predict CH_4_ emissions from Italian lactating Holstein dairy cows. The data set included individual observations from 3 in vivo studies (Gislon et al., 2020b; Pirondini et al., 2015; and Colombini et al., 2015), with 32, 16, and 18 observations, respectively. The experiments were carried out at the University of Milan Research Center, in the Po plain (Milan, Italy; 45°30′N, 9°1′E) where the climate of the region is mainly humid subtropical according to the Geiger–Kӧppen climate classification. The diets fed in each experiment were representative of typical diets fed in the region and were based on the following forages: alfalfa and grass silages, alfalfa and grass hays, wheat silage, corn silage, sorghum forage silage, and sorghum grain whole plant silage. Methane emissions were measured through individual open-circuit respiration chambers, and the DE was determined in vivo by total feces collection. Further details are reported in the above-cited studies, but briefly the air flow through the chambers was measured using diaphragm flow-meters at ambient temperature, pressure, and relative humidity. The air flux was then referred to standard conditions (0°C, 1 atm, relative humidity = 0) by a calculation based on temperature, pressure, and relative humidity measured in the outgoing air entering the flow-meter.

Before each respiration chamber cycle, calibrations were made with a certified reference gas with methane concentrations close to the maximum values detectable by the analyzer (2,000 ppm of CH_4_) and pure N_2_ to calibrate the minimum value. The calculation of CH_4_ energy (kJ) was determined by multiplying CH_4_ production in liters at standard conditions by 39.5388. The production of CH_4_ in grams was obtained multiplying the CH_4_ production (in liters at standard conditions) by 0.71682. Five predicting equations were applied based on different Y_m_ and DE values. The first equation (**IPCC06**) was based on IPCC 2006 (IPCC, 2006b) Tier 2 (IPCC Table 10.12) with Y_m_ = 6.5% and DE = 70%. Two other equations were based on IPCC 2019 (IPCC, 2019a) Tier 2 as follows: (1) the average Y_m_ (5.7%) and DE fixed at 70% were used to calculate the ratios of net energy available in a diet for maintenance and growth to DE consumed (equations 10.14 and 10.15, respectively) and to calculate the gross energy (**GE**) requirements (equation 10.16; **1YM**) or (2) the average Y_m_ (5.7%) and the replacement of fixed DE with the average in vivo digestibility from the experiments (**1YMIV**). The final 2 equations were based on IPCC 2019 (IPCC, 2019a; Table 10.12) as follows: for diets with NDF ≤35%, the value 5.7% of Y_m_ was used, whereas for diets with NDF >35%, the value 6.0% of Y_m_ was applied; for DE a fixed digestibility at 70 (**2YM**) or the average in vivo digestibility from the experiments was used (**2YMIV**). The emission factor was calculated from IPCC 2019 (equation 10.21) on the basis of the estimate of GE intake since according to IPCC (2019a) “the Tier 2 estimate of gross energy is the preferred method” for the calculation of the emission factor.

An IPCC Tier 2 model for cows fed typical Mediterranean diets (**MED**) was also proposed using the average value of Y_m_ (5.58) and the average DE values of 69.9 or 64.8% for silage- or hay-based diets respectively obtained from the in vivo experiments, as described in a following section of the paper. The MED was validated on an independent data set based on individual cow observations from the study of Enriquez-Hidalgo et al. (2020) conducted in a central region of Chile characterized by a Mediterranean climate with hot summers. The Chilean study evaluated 2 diets, one similar to diets typically fed in Italy with a forage basis including corn silage and alfalfa hay, and the other not typically Mediterranean, based on corn silage and a mixture of fresh annual ryegrass and berseem clover (**MIX**). The CH_4_ emission was measured with the SF_6_ technique. For this data set, the 1YM and MED equations were applied. It was not possible to apply the other equations because DE was not measured and the 2 diets had NDF <35% DM.

The predictive equations for CH_4_ emission were evaluated according to Tedeschi (2006). The root mean square prediction error (**RMSPE**) was decomposed into error due to mean bias (**MB**), error due to slope bias (**SB**), and error due to random bias (**RB**; Bibby and Toutenburg, 1977).

The concordance correlation coefficient (**CCC**) was calculated according to Lin (1989) as follows:

CCC = r × Cb,

where r is the Pearson correlation coefficient and Cb is a bias correction factor (a measure of accuracy), calculated as follows:

Cb = 2/(V + 1/V + µ^2^),

where V is a measure of scale shift (i.e., the change in standard deviation between predicted and observed values), and μ is a measure of location shift. V and μ were calculated as below:

V = SD_O_/SD_P_,

µ = (M_O_ − M_P_)/(SD_O_ × SD_P_)^1/2^,

where M_O_ and M_P_ are the means of observed and predicted values, respectively, and SD_O_ and SD_P_ are the standard deviations of observed and predicted values, respectively.

Data were analyzed using the Proc Mixed procedure of SAS 9.4 (SAS Institute Inc.) with the model Y_jkl_ = μ + D_j_ + S_k_ + COW_l_(S_k_) + ε_jkl_, where Y_jkl_ is the dependent variable (DE and Y_m_); μ is the overall mean; D_j_ is the diet effect (j = 1, 5); S_k_ is the random study effect (k = 1, 3); COW_l_ is the random animal effect nested within study (l = 1, 18), and ε_jkl_ is the residual error.

The results of IPCC predictions are in Table 1. The RMSPE (%) was similar among models (on average 10.5); all values were <13%, and according to Kaewpila and Sommart (2016), a model may be considered inadequate when RMSPE is higher than 16.0%. The RMSPE reported by Niu et al. (2018, 2021) were comparable to those obtained in the present study.

**Table 1:** Results of RMSPE and CCC analysis for CH_4_ production for the individual lactating cow database predicted using IPCC06 (Y_m_ = 6.5%, DE = 70%), or using IPCC (2019a) as follows: 1YM (Y_m_ = 5.7%, DE = 70%), 1YMIV (Y_m_ = 5.7%, DE measured in vivo), 2YM (Y_m_ = 5.7 or 6.0% depending on diet NDF, DE = 70%), or 2YMIV (Y_m_ = 5.7 or 6.0% depending on diet NDF, DE measured in vivo)^1^

Model	Diet NDF (%)	DE (%)	Y_m_ (%)	CH_4_ (g/d)	RMSPE (%)	MB (%)	SB (%)	RB (%)	CCC	r	Cb	V	μ
In vivo	—	—	—	381	—	—	—	—	—	—	—	—	—
IPCC06	—	70	6.5	428	12.6	59.7	2.96	38.0	0.400	0.630	0.636	1.18	−1.05
1YM	—	70	5.7	370	9.40	9.69	1.88	89.9	0.579	0.630	0.919	1.35	0.296
1YMIV	—	69.8	5.7	377	10.1	0.968	19.2	81.5	0.569	0.572	0.995	1.03	0.091
	—	64.8	5.7	377	10.1	0.968	19.2	81.5	0.569	0.572	0.995	1.03	0.091
2YM	<35	70	5.7	377	9.27	0.928	2.46	98.2	0.536	0.572	0.937	1.42	0.095
	>35	70	6.0	377	9.27	0.928	2.46	98.2	0.536	0.572	0.937	1.42	0.095
2YMIV	<35	69.8	5.7	384	11.2	0.614	28.2	72.8	0.488	0.490	0.997	0.968	−0.079
	>35	64.8	6.0	384	11.2	0.614	28.2	72.8	0.488	0.490	0.997	0.968	−0.079

1 DE = energy digestibility; 69.8% for silage-based diets, 64.8% for hay-based diets. Y_m_ = conversion factor of gross energy intake into enteric CH_4_ energy. RMSPE = root mean square prediction error expressed as a percentage of the observed mean. MB = error due to bias, as a percent of total RMSPE. SB = error due to slope, as a percent of total RMSPE. RB = error due to random bias as a percent of total RMSPE. CCC = concordance correlation coefficient, where CCC = r × Cb. r = Pearson correlation coefficient. Cb = bias correction factor. V = scale shift. μ = location shift. IPCC06 = first equation based on Intergovernmental Panel on Climate Change 2006 (IPCC, 2006b).

The IPCC06 was the less accurate model (MB = 59.7%; Cb = 0.636) with an overestimate of CH_4_ emission (428 vs. 381 g/d, respectively, for IPCC06 and in vivo). Overestimation of IPCC06 was also found by Appuhamy et al. (2016) for North American diets. Similarly, Jiménez et al. (2021) showed that the calculation of CH_4_ using specific emission factors for tropical climate regions is better than the IPCC 2006 default emission factors, which overestimated CH_4_ production. The Y_m_ value used in the IPCC06 equation (6.5%) explains the overestimation. To the best of our knowledge, only one study (Benaouda et al., 2020) applied the IPCC refinement (IPCC, 2019a) to estimate cattle enteric CH_4_ in Latin America, and the results showed that the new factors of IPCC (2019a) made a substantial improvement in the prediction compared with the previous IPCC Tier 2 (IPCC, 2006b). Given the importance of Y_m_, for large regions such as Europe, using specific values that better represent the characteristics of local production systems is advisable. For example, a recent study showed that the predicted Y_m_ ranged from 6.22 to 6.72% for Norway (Niu et al., 2021), whereas in the Netherlands, a Tier 3 approach used a predicted Y_m_ ranging from 5.88% to 6.07% (Bannink et al., 2011).

The 1YM model was the second least accurate model (MB = 9.69; Cb = 0.919) with underestimated emissions (370 vs. 381 g/d). The underestimation was mainly due to the hay-based diets: for these diets, the use of a fixed DE (70%) rather than the lower in vivo value (64.8%) reduced the predicted GE requirement (Equation 10.16) and hence the related CH_4_ emission.

The models 2YMIV, 2YM, and 1YMIV all have a MB below 1% and a Cb > 0.93. Using the in vivo DE improved the accuracy of the models, as confirmed also considering the V values. The models that best predicted the in vivo variability (V close to 1) were the ones using DE measured in vivo (V = 1.03 and 0.968 for 1YMIV and 2YMIV, respectively). The importance of DE as a key factor to estimate CH_4_ emissions has long been known, although it is not an easily measurable parameter. Other studies suggested the use of OM digestibility to predict CH_4_ emissions (Ramin and Huhtanen, 2012; Bell et al., 2016). However, models that include OM digestibility had lower precision and accuracy than those based on DE (Benaouda et al., 2019).

In contrast, using in vivo DE values decreased the model precision (SB = 19.2 and 28.2% and r = 0.572 and 0.490 for 1YMIV and 2YMIV, respectively). The most precise model was 1YM (SB = 1.88%; r = 0.63) followed by IPCC06 (SB = 2.96; r = 0.63) and 2YM (SB = 2.46; r = 0.572). Given the differences in precision and accuracy, the CCC parameter has been suggested to simultaneously account for accuracy and precision (Tedeschi, 2006) and the CCC evaluation showed the highest (best) value for the model 1YM (0.579) followed, with a similar value, by 1YMIV (0.569).

The in vivo values of DE and Y_m_ for cows fed Mediterranean diets are shown in Table 2. The mean DE varied across study and diet (*P* < 0.001), with the hay diet studied by Gislon at al. (2020b) having significantly lower DE values than all other studies and diets except the corn silage diet within the study of Colombini et al. (2015). Several studies (i.e., Broderick, 1995; Gislon et al., 2020b) reported a higher DMI for cows fed hay-based diets than silage diets, and increased intake may increase passage rate, thus decreasing the DE (Gislon et al., 2020b). Moreover, the hay-based diet was characterized by a low NDF digestibility, further decreasing energy digestibility. Overall, there is scarce information about the DE measured in vivo of cows fed hay diets. For example, Klevenhusen et al. (2011) reported a DE of 65.3% in lactating dairy cows fed a hay-based diet, a value comparable to the Italian hay diet data set.

**Table 2:** Digestible energy (DE; %) and Y_m_ (%), and dietary NDF and starch (% DM) determined in cows fed Mediterranean diets with a different forage basis^1^

Item	Study							SEM	*P*-value
	Gislon et al. (2020b)				Pirondini et al. (2015)	Colombini et al. (2015)			
	CS	AGS	WS	Hay	CS	CS	SS		
DE	73.6^a^	72.6^ab^	70.3^bc^	64.8^e^	68.6^cd^	65.2^de^	68.9^c^	1.30	<0.001
Y_m_	5.67^a^	5.92^a^	5.82^a^	5.68^a^	5.41^ab^	5.05^b^	5.52^a^	0.208	0.007
NDF	32.8	27.1	37.7	36.6	33.0	36.6	36.4		
Starch					25.9	26.5	25.9		

a–e Least squares mean values within a row with different superscripts differ significantly at *P* < 0.05.

1 CS = diet based on corn silage; AGS = diet based on alfalfa and grass silage; WS = diet based on wheat silage; Hay = diet based on hay; SS = diet based on sorghum silage; Y_m_ = conversion factor of gross energy intake into enteric CH_4_ energy.

The mean Y_m_ also varied across study and diet (*P* = 0.007), although to a lesser extent and with no individual forage standing out as being different to the others. The IPPC refinement Tier 2 (IPCC, 2019a) for the European data set (milk yield >8,500 kg) assumes a Y_m_ value of 6.0%. This value is higher than the average value (5.58%) observed in the data set of the present study. The most studied forage type for Europe is grass silage, followed by corn silage and fresh forage; diets based on grass silage are expected to have higher Y_m_ values than diets based on corn silage due to the higher starch content of the latter, which increases the dietary starch concentration (Hassanat et al., 2013; Benchaar et al., 2014). Based on these results, Mediterranean coefficients of Y_m_ and DE appropriate for the MED equation were proposed as follows: Y_m_ = 5.58% (average of Y_m_ values for all diets, since no effect of diet was observed) and DE = 69.9 and 64.8% for silage- and hay-based diets, respectively.

The cross-validation results on the independent data set (Enriquez-Hidalgo et al., 2020) are in Table 3. To highlight potential differences between equations, both a typical Mediterranean-type corn silage and hay-based diet, and a corn silage and a mixture of fresh annual ryegrass and berseem clover diet (MIX) were cross-validated. The average RMSPE (28.9%) was higher than that of the Italian data set (10.5%). Part of the reason for this discrepancy could be the method used to measure CH_4_ emission (respiration chambers for MED model, SF_6_ in the data set for cross-validation). Moreover, the SF_6_ technique collects the animal breath CH_4_ emissions, but not the rectum CH_4_ emissions, and this can partially explain the differences observed between the in vivo and the model estimations. Enriquez-Hidalgo et al. (2020) modified the SF_6_ technique according to Deighton et al. (2014), to reduce the variability of CH_4_ yield estimation between cows, obtaining an accuracy similar to respiration chambers, although respiration chambers remain the gold standard method for measuring CH_4_ emission (Garnsworthy et al., 2019).

**Table 3:** Results of RMSPE and CCC analysis on CH_4_ production for an independent individual lactating cow database derived from Enriquez-Hidalgo et al. (2020) using IPCC (2019a) 1YM (Y_m_ = 5.7%, DE = 70%) or the Tier 2 coefficients derived from the present study (MED, Y_m_ = 5.58%; DE = 69.9%)^1^

Model	CH_4_ (g/d)	RMSPE (%)	MB (%)	SB (%)	RB (%)	CCC	r	Cb	V		μ
Corn silage and alfalfa hay diet											
In vivo	396										
1YM	405	30.8	0.547	52.3	47.1	0.492	0.858	0.573	3.16		−0.103
MED	397	31.5	0.009	53.2	46.7	0.485	0.856	0.565	3.22		−0.013
Corn silage and fresh annual ryegrass/berseem herbage (MIX)											
In vivo	334										
1YM	366	26.7	9.70	1.03	90.3	0.169	0.277	0.610	2.64		−0.511
MED	358	26.7	5.78	0.904	93.3	0.172	0.277	0.621	2.69		−0.392

1 DE = energy digestibility; 69.8% for silage-based diets, 64.8% for hay-based diets. Y_m_ = conversion factor of gross energy intake into enteric CH_4_ energy. RMSPE = root mean square prediction error expressed as a percentage of the observed mean. DE = digestible energy. MB = error due to bias, as a percent of total RMSPE. SB = error due to regression, as a percent of total RMSPE. RB = error due to random bias, as a percent of total RMSPE. CCC = concordance correlation coefficient, where CCC = r × Cb. r = Pearson correlation coefficient. Cb = bias correction factor. V = scale shift. μ = location shift.

The 1YM and MED had a higher (best) accuracy (evaluated in terms of MB) and CCC for corn silage and hay diet, rather than for the MIX diet. The predicted CH_4_ was higher than the in vivo value but differed between diets with a greater overestimation (evaluated in terms of µ) for the MIX diet (µ = −0.452, on average of 1YM and MED) than the corn silage + hay diet (−0.058, on average of 1YM and MED). The presence in the MIX diet of berseem clover, a forage containing natural substances able to reduce methanogenesis (Enriquez-Hidalgo et al., 2020), partially explains the IPCC method's overestimation of CH_4_ emissions for this diet compared with direct in vivo measurement. In contrast, the predicted CH_4_ emission on the corn silage diet using our proposed Mediterranean coefficients was very similar to that measured in vivo (396 and 397 g/d, respectively, for in vivo and MED).

The RB value was higher for the MIX diet (91.8%, on average) than for the corn silage and hay diet (46.9%, on average); however, the r values were very low for the prediction of the MIX diet (on average, 0.277) compared with the corn silage and hay diet (on average, 0.857). The high RB for the MIX diet is explained by the lower values of predicted standard deviation compared with the in vivo and the low r value.

In conclusion, the study showed that IPCC 2019 predicts CH_4_ emission accurately and then it can be used as a tool for the prediction of CH_4_ emissions for inventories; however, for Mediterranean diets specific values of Y_m_ and DE may be preferable, especially for hay-based diets.
